# Natural Clay as a Low-Cost Adsorbent for Crystal Violet Dye Removal and Antimicrobial Activity

**DOI:** 10.3390/nano11112789

**Published:** 2021-10-21

**Authors:** Ali Q. Alorabi, Mallick Shamshi Hassan, Mohammad Mahboob Alam, Sami A. Zabin, Nawaf I. Alsenani, Neazar Essam Baghdadi

**Affiliations:** 1 Chemistry Department, Faculty of Science, Albaha University, P.O. Box 1988, Albaha 65799, Saudi Arabia; samizabin@gmail.com (S.A.Z.); n.alsenani@bu.edu.sa (N.I.A.); 2 Center of Nanotechnology, King Abdul Aziz University, P.O. Box 80216, Jeddah 21589, Saudi Arabia; Nebaghdadi@kau.edu.sa

**Keywords:** natural micro and nanoclay, crystal violet, adsorption, isotherm, biological activity

## Abstract

This investigation aimed at evaluating the efficiency of micro and nanoclays as a low-cost material for the removal of crystal violet (CV) dye from an aqueous solution. The impacts of various factors (contact time, pH, adsorbent dosage, temperature, initial dye concentration) on the adsorption process have been taken into consideration. Six micro and nanoclay samples were obtained by treating clay materials collected from different locations in the Albaha region, Saudi Arabia. Out of the six tested micro and nanoclays materials, two (NCQ1 and NCQ3) were selected based on the highest adsorption efficiency for complete experimentation. The morphology and structure of the selected micro and nanoclay adsorbents were characterized by various techniques: SEM-EDX, FTIR, XRF, XRD, and ICP-MS. The XRF showed that the main oxides of both nanoclays were SiO_2_, Al_2_O_3_, Fe_2_O_3_, K_2_O, CaO, and MgO, and the rest were impurities. All the parameters affecting the adsorption of CV dye were optimized in a batch system, and the optimized working conditions were an equilibrium time of 120 min, a dose of 30 mg, a temperature of 25 °C, and an initial CV concentration of 400 mg/L. The equilibrium data were tested using nonlinear isotherm and kinetic models, which showed that the Freundlich isotherm and pseudo-second-order kinetics gave the best fit with the experimental data, indicating a physico-chemical interaction occurred between the CV dye and both selected micro and nanoclay surfaces. The maximum adsorption capacities of NCQ1 and NCQ3 adsorbents were 206.73 and 203.66 mg/g, respectively, at 25 °C. The thermodynamic factors revealed that the CV dye adsorption of both micro and nanoclays was spontaneous and showed an exothermic process. Therefore, the examined natural micro and nanoclays adsorbents are promising effective adsorbents for the elimination of CV dye from an aqueous environment.

## 1. Introduction

Synthetic organic dyes released from different industries are one of the most significant sources of water pollution and they are of longstanding concern to human beings and other living organisms due to their adverse effects [1,2]. On account of their toxicity, they must be removed from water resources in order to save living organisms [3]. Crystal Violet (CV; C_25_N_3_H_30_Cl), which is a cationic dye named triphenylmethane, has been widely used in wool and nylon dyeing, manufacturing wax dyes, and printing inks [4] and has been proved to be a toxic, carcinogenic agent, causing severe eye irritation, kidney failure, and skin problems, etc. [5]. Moreover, it may persist in the environmental for a long period [6,7].

Therefore, serious efforts have been devoted by researchers in recent decades to remove dyes from industrial effluents and purify wastewater using different technologies including physiochemical and biological methods such as reverse osmosis [8], photocatalytic degradation [9], chemical oxidation [10], biological degradation [11], electrochemical treatment [12], and adsorption [13,14]. Among these techniques, adsorption stands out as a highly competent technology for the removal of pollutants from wastewater due to its simple, easy, cost-effective, efficient, and eco-friendly techniques [15,16].

Various inorganic and organic adsorbents were reported for the removal of CV dye, such as an activated carbon-based adsorbent that showed good performance in colored wastewater treatment, but the high costs and strict preparation conditions hinder the application of these adsorption materials [17]. The cost, toxicity, availability, and reusability are the parameters important for the selection of adsorbents in wastewater treatment. Therefore, many studies have been carried out to search for low-cost adsorbents derived from natural materials to efficiently remove CV dye. Among various adsorbents, natural clay is abundantly present in nature in large quantities as a low-cost, accessible, non-toxic, material and has a high potential for ion exchange for charged pollutants, which has the potential for use in wastewater treatments. Clays are mostly comprised of alumina–silicates, which have a layered structure, and are one of the most natural materials that have been applied for almost all types of pollutant adsorption from wastewater [18]. Various types of natural clay structures have been applied to water treatment, such as K10-Montmorillonite [19], Montmorillonite Clay Mineral [20], pyrophyllite [6], bentonite [21], and mixed clay (kaolinite/pyrophyllite) [22]. Nanoclay minerals are characterized by a large specific surface area and high adsorption capacity that provide them chemical stability and high structural features for the removal of contaminants [23].

In addition to the adsorption character of nanoclays, it was reported that natural nanoclays have antimicrobial action and could be used in crops and food packaging coatings and as encapsulation matrices [24,25].

The present work aims to investigate the feasibility of using natural Saudi micro and nanoclays as a low-cost adsorbent prepared from natural clay materials collected from the Albaha region, in the southwestern part of Saudi Arabia. The targeted clay materials, to the best of our knowledge, have not been investigated before as adsorbents for the elimination of crystal violet dye or as antimicrobial agents. The targeted natural Saudi micro and nanoclays were applied for the elimination of crystal violet molecules from aqueous solutions, and we have also investigated their antimicrobial activity against standard bacterial and fungal strains. The examined micro and nanoclays were characterized using different techniques such as SEM-EDX, FTIR, XRF, XRD, and ICP-MS. The impact of key operational parameters such as contact time, pH solution, initial CV concentration, adsorbent dosage, and temperature on the adsorption of CV dye was determined. The kinetics, isotherm, equilibrium, and thermodynamics of the adsorption process were also investigated. The concentrations of crystal violet molecules on the tested micro and nanoclays adsorbents were examined using UV/Vis Spectrophotometry.

## 2. Materials and Methods

### 2.1. Chemicals and Instrumentation

Crystal violet (CV), methylene blue (MB), and malachite green (MG) were procured from Sigma Aldrich (St. Louis, MO, USA). Hydrochloric acid (HCl, 36%), sodium hydroxide (NaOH, ≥97%), nitric acid (HNO_3_, 68.0–70.0%), and sodium chloride (NaCl, ≥99%) were obtained from BDH, England (Poole, Doorset, UK). The morphology and elemental composition of the dried micro and nanoclays adsorbents were performed using scanning electron microscopy (SEM) and energy-dispersive X-ray (EDX) (JEOL 7600F, Tokyo, Japan). FTIR spectra of the tested micro and nanoclays adsorbents before and after CV dye adsorption were recorded on the Thermo Scientific FTIR Spectrometer (Nicolet iS50 FTIR, Madison, WI, USA) in the range 4000–400 cm^−1^. The X-ray diffraction (XRD) analysis of the micro and nanoclays adsorbents was accomplished using a Shimadzu XRD-6000, (Tokyo, Japan) diffractometer. A UV-vis spectrophotometer was utilized to determine the concentrations of dyes on the micro and nanoclay adsorbents. The chemical composition of micro and nanoclay adsorbents was obtained by XRF. Inductively Coupled Plasma-Optical Emission Spectroscopy (ICP-OES) (Thermo Scientific, Weltham, MA, USA) was applied for the analysis of micro and nanoclay adsorbents.

### 2.2. Preparation of the Micro and Nanoclays

Six clay mineral samples (NCQ1, NCQ3, NCQ4, NCQ5, NCR1 (Shahba Forest), and NCY1 (Alboaithah)) were collected from different locations of the Albaha region, Saudi Arabia. The collected natural Saudi clay samples were first purified by dispersion in water and decanted two times. The samples were then treated with 0.1M CH_3_COOH to remove carbonates and with 35% H_2_O_2_ to remove minerals and organic impurities. Finally, the nanoclay was rinsed with deionized water to remove the traces of acetic acid and hydrogen peroxide [26]. The dried clay samples were crushed and then passed through a 1 mm mesh sieve to obtain fractions of a narrow size. Then, 20 g of soil was dispersed with 100 mL of distilled water using ultrasonication for 1 h. After that the soil suspension was added to a cylinder containing 900 mL of deionized (D.I.) water, left for 2 h, then around 800 mL of supernatant was collected in a cylinder, and the remaining 200 mL of clay suspension was transferred to a cylinder containing 800 mL of D.I. water and left for 24 h. This procedure was repeated thrice. The supernatant of clay collected in the previous step was dried using an oven at 60 °C for 3 days. Finally, 1 g dried clay powder was suspended in a 100 mL aqueous solution of 1 M NaCl under continuous stirring for 24 h, then separated by centrifugation. The clay was washed several times by D.I. water and dried in an oven. The sample was crushed to obtain fine powder and stored in a desiccator for analysis and further use in adsorption and biological activity experiments. The same procedure was applied for the other five clay mineral samples.

### 2.3. Adsorption Experimentation

First of all, we screened all the six clay samples collected from different parts of the Albaha region, Saudi Arabia (NCQ1, NCQ3, NCQ4, NCQ5, NCR1, and NCY1) for their adsorption ability for the elimination of three cationic dyes, MG, MB, and CV, from aqueous solutions. According to the adsorption experimental results, we selected NCQ1 and NCQ3 as the best adsorbents from the tested micro and nanoclays. To evaluate the influence of different factors, such as adsorbent dose, initial MG, MB, and CV concentration, solution pH, contact time, and temperature, on CV dye adsorption on both micro and nanoclay NCQ1 and NCQ3 adsorbents, batch adsorption experiments were used. A 250 mL conical flask containing 50 mL of 20 mg/L different dyes with 30 mg of the introduced adsorbents was shaken at 100 rpm at 24 h. Then, the sample was centrifuged at 4000 rpm for 15 min to remove the adsorbent. After that, the residual CV, MB, and MG dye ion concentrations were measured by UV-visible spectra at λ_max_ (590 nm), (663 nm), and (617 nm), respectively. The pH effect was studied at pH values ranging 4–9 after adjusting for the addition of 0.1 M HCl and 0.1 M NaOH solution and was recorded using a table desktop pH meter. The influence of the contact time, initial CV concentration, adsorbent dosage, and temperature on the CV adsorption on both selected micro and nanoclay adsorbents were performed in the range (5–480 min), (20–500 mg/L), (10–100 mg), and (25–45 °C), respectively. The adsorption capacity *q_e_* (mg/g) and percentage of adsorption for both micro and nanoclay adsorbents were expressed using Equations (1) and (2), respectively.

(1) $$\;q_{e}=\left(C_{o}-C_{e}\right)\frac{V}{m}$$

(2) $$\%\;\mathrm{Adsorption}=\frac{C_{o}-C_{e}}{C_{o}}\times 100$$

where *C_o_* is the initial dye concentration, *C_e_* (mg/L) is the residual dye concentration at time *t* (min), *m* (g) is the mass of adsorbents, and *V* (L) refers to the volume of dye solutions.

### 2.4. Antimicrobial Activity

All the antimicrobial in vitro experiments were performed at the Blood bank and Microbiology Laboratory in Bisha city, Kingdom of Saudi Arabia. The discs of the clay samples were prepared by dissolving 10% polyvinyl alcohol (PVA) in water; the mixture of PVA and water was stirred at room temperature for 24 h, and then 5% clay were added to this mixture. The combined mixture was further stirred for two hours and then poured onto glass petri dishes and kept overnight at room temperature to dry. After drying, the discs were prepared from the PVA film by a 6 mm borer. In a similar way, 1% of ciprofloxacin was used as a positive control and a disc of 10% PVA in water was used as a negative control. The clinical strains *E. coli*, *S. aureus*, *E. faecalis*, *K. pneumonia*, and *C. albicans* were used throughout all antimicrobial experiments. The susceptibility test was carried out using the reported disc diffusion method [27]. Each microbial strain was suspended in Mueller Hinton (MH) (Difco, Detroit, MI, USA) broth and diluted to approximately 10^6^ colony-forming units (cfu)/mL. They were ‘‘flood-inoculated’’ onto the surface of MH agar and then dried. Antimicrobial activity was evaluated by measuring the zone of inhibition (in mm) against the tested organism.

## 3. Results

### 3.1. Selectivity Investigation of Micro and Nanoclay Adsorbent

**Figure 1:**
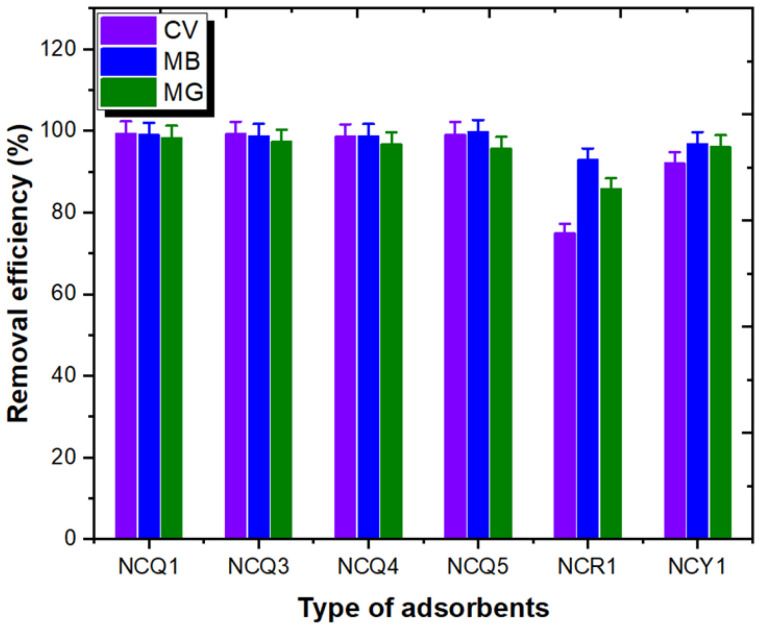
Adsorption selectivity experiments with dye solutions.

To select the most appropriate micro and nanoclay for MG, CV, and MB adsorption from aqueous media, six different natural Saudi micro and nanoclay samples, NCQ1, NCQ3, NCQ4, NCQ5, NCR1, and NCY1, were examined, and the results obtained are shown in Table 1 and Figure 1. The experimental observations displayed that the adsorption efficiency of the MG, MB, and CV dyes was greater than 90% for all tested micro and nanoclay materials except NCR1, which showed ra emoval efficiency of 75% in the case of CV dye and 86% for the MG dye. The adsorption efficiency observed was in the order of NCQ1 ≥ NCQ3 > NCQ5 > NCQ4 > NCY1 > NCR1. Therefore, the highest dye adsorption efficiency values were seen when using NCQ1 and NCQ3 micro and nanoclays (Table 1). Moreover, the maximum dye removal efficiency was observed in the case of the CV dye with 99.34% and 99.25% when using NCQ1 and NCQ3, respectively. Accordingly, NCQ1 and NCQ3 micro and nanoclay samples were selected for detailed CV adsorption experimentation in this investigation.

### 3.2. Characterization of the Micro and Nanoclay Adsorbents

#### 3.2.1. FTIR Spectra Analysis

**Figure 2:**
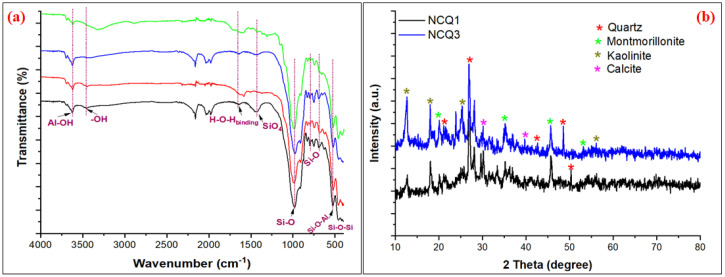
(**a**) FT-IR spectra (**b**) XRD pattern of NCQ1 and NCQ3 adsorbents.

Figure 2a represents the FTIR spectra for both NCQ1 and NCQ3 adsorbents before and after CV dye adsorption. It is observed that the FTIR spectra for both selected micro and nanoclay adsorbents before the CV dye adsorption was the same. Peaks appeared at 3626 cm^−1^, which can be assigned to the inner surface of the stretching Al-OH bond [28]. The peak at 3456 cm^−1^ is due to the presence of the outer surface of the O-H group. A weak broad band due to O-H bending of the water molecule and a strong peak of siloxane Si-O-Si (out of and within the plane) were observed at 1641 cm^−1^ and 988 cm^−1^, respectively [29,30]. The broad band observed at 1426 cm^−1^ is due to the presence of calcite [31] or the vibrational modes of SiO_4_ tetrahedron in montmorillonite [30]. The peaks that appeared in the 988–459 cm^−1^ region may be attributed to the micro and nanoclay minerals and silicon-containing groups (Al-O-Si and Si-O-Si) [32,33,34]. The peak observed in the lower range at 914 cm^−1^ is assigned to the stretch vibration of the inner surface of the –OH group. The peak at 792 cm^−1^ was specific to Si-O bending bands of quartz [31]. The peaks that appeared at 464 cm^−1^ and 524 cm^−1^ were characteristic of Si-O-Si and Al-O-Si bending vibrations, respectively [35]. After the adsorption of CV, some peaks disappeared and other peaks showed a decrease in intensity and a shift in position. For example, the peak due to the–OH bond that appeared at 3456 cm^−1^ was shifted to a lower frequency after CV adsorption and appeared at 3452 cm^−1^ and 3340 cm^−1^ when using NCQ1 and NCQ3, respectively. This shift may be explained by the formation of hydrogen bonds between the CV dye and the NCQ1 and NCQ3 surfaces. The peaks of Si-O-Si and Si-O-Al groups that appeared in the range 988–524 cm^−1^ decreased to a lower frequency (978–518 cm^−1^) after CV adsorption due to the interaction of the CV dye molecules with Si-O-Si and Si-O-Al groups on the surface of both NCQ1 and NCQ3 adsorbents [36]. Furthermore, the peak of O-H bending due to water molecules decreased in intensity and shifted to a lower frequency (1633 cm^−1^) [37].

#### 3.2.2. XRD Analysis

NCQ1 and NCQ3 micro and nanoclays refer to a group of expanding clay minerals and are mainly a mixture of quartz, montmorillonite, kaolinite, and calcite as presented in Figure 2b. The structure was proved by XRD analysis, which showed that both NCQ1 and NCQ3 adsorbents have characteristic peaks of quartz at 20.23°, 26.93°, 42.45°, and 50.24° [1,38,39]. In addition, the peaks at 2Ɵ of 20.07°, 35.08°, 45.74°, and 53.8° correspond to the interlayer spacing of the montmorillonite structure [40,41,42]. The characteristic peaks for calcite appeared at 2Ɵ 29.8° and 39.96° [43]. The peaks that appeared at 12.32°, 25.16°, and 55.92° are due to kaolinite minerals [44,45]. These results were consistent with those reported by Gong et al. (2021) [39]. Therefore, both NCQ1 and NCQ3 adsorbents are composed of clay minerals: Quartz, montmorillonite, kaolinite, and calcite.

#### 3.2.3. XRF Compositional Analysis

**Figure 3:**
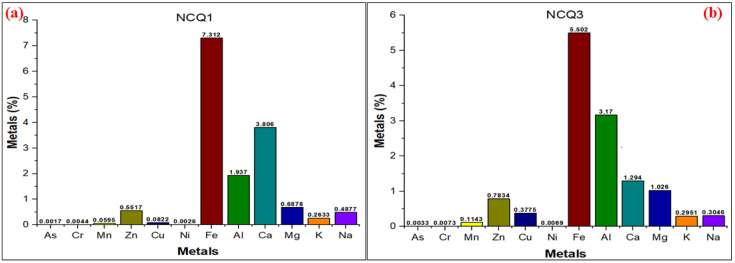
(**a**) ICP-MS of NCQ1 (**b**) and NCQ3 adsorbents.

The compositional analysis of NCQ1 and NCQ3 adsorbents was measured by the XRF technique, and the obtained results are presented in Table 2 and Table 3. The analysis revealed that the highest weight percentages (wt%) among the oxides present in NCQ1 and NCQ3 adsorbents were of SiO_2_ (46.84%) and (50.76%), respectively, followed by Al_2_O_3_ (28.32%) and Fe_2_O_3_ (13.59%) for NCQ1 and (28.63%) and (10.49%) for NCQ3, respectively. Comparably, the amount of SiO_2_ (50.76%) was higher in the NCQ3 micro and nanoclay sample. The high quantities of SiO_2_ and Al_2_O_3_ detected in the two samples revealed that the NCQ1 and NCQ3 adsorbents belong to aluminosilicate. Some other oxide constituents (such as K_2_O, CaO, and SO_3_) exist but in small percentages. The mass ratio of SiO_2_/Al_2_O_3_ was found to be 1.65% and 1.77% for both NCQ1 and NCQ3 adsorbents, respectively, indicating that the main constituent of both tested adsorbents is quartz. Measurements of the elemental concentrations in NCQ1 and NCQ3 adsorbents were determined using inductively coupled plasma mass spectrometry (ICP-MS) as shown in Figure 3a,b. The observations indicated the presence of major and minor elements as revealed by the metal % values.

#### 3.2.4. Morphological Image Analysis and Elemental Composition

**Figure 4:**
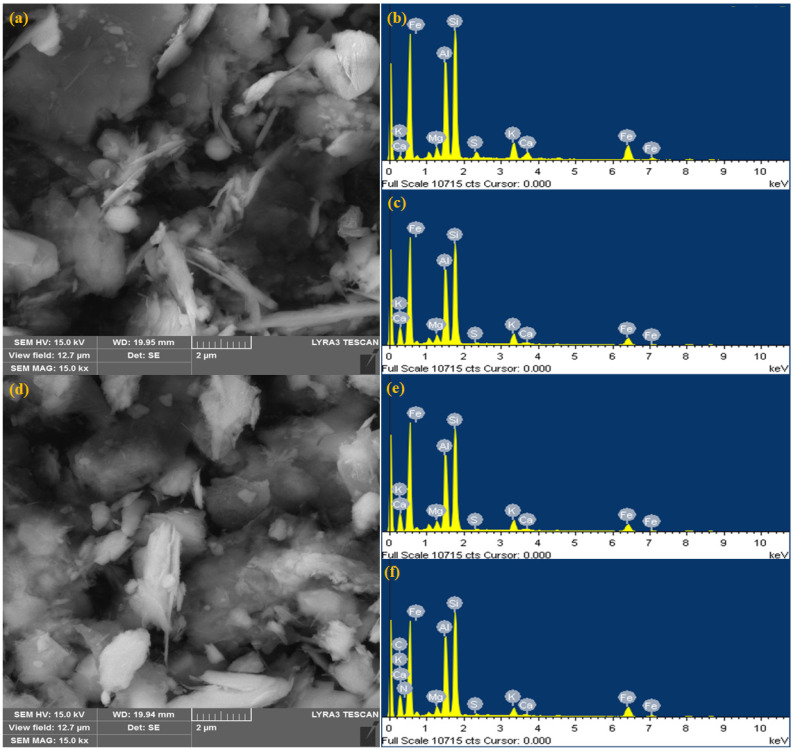
SEM image (**a**), and EDX analysis of NCQ1 (**b**), SEM image (**d**), and EDX analysis of NCQ3 (**e**), EDX analysis after CV dye adsorption onto NCQ1 (**c**), and NCQ3 (**f**) adsorbents.

The morphology of the selected micro and nanoclay adsorbents was determined utilizing the SEM microscope technique. The morphological images for NCQ1 and NCQ3 with different magnifications are presented in Figure 4a,d, respectively. It was seen that the shape of both micro and nanoclay particles seems to be spherically aggregated and compacted platelets with sizes between 500 nm to several micrometers. The elemental composition of the micro and nanoclays before and after the adsorption process was examined using the EDX technique. Figure 4b,e shows the elemental analysis of NCQ1 and NCQ3 adsorbents before the adsorption process of the CV dye. Figure 4b,e suggests the presence of major elements, namely Si, Al, Fe, K, and Ca, in both micro and nanoclay materials. These results are in agreement with XRF analysis results as discussed above. The appearance of two new peaks for C and N after adsorption in the EDX analysis of both NCQ1 and NCQ3 adsorbents confirmed that the CV dye molecules were adsorbed successfully onto both NCQ1 and NCQ3 adsorbent surfaces (Figure 4c,f). On comparing the EDX analysis for both NCQ1 and NCQ3 adsorbents before and after the CV dye adsorption process, it is observed that K^+^ and Ca^2+^ ions were decreased in both adsorbents after cationic CV adsorption. The new peaks that appeared after adsorption for N and C may be a consequence pf the exchange of cationic CV molecules by K^+^ and Ca^2+^ cations.

### 3.3. Adsorption Performance of the Micro and Nanoclays

**Figure 5:**
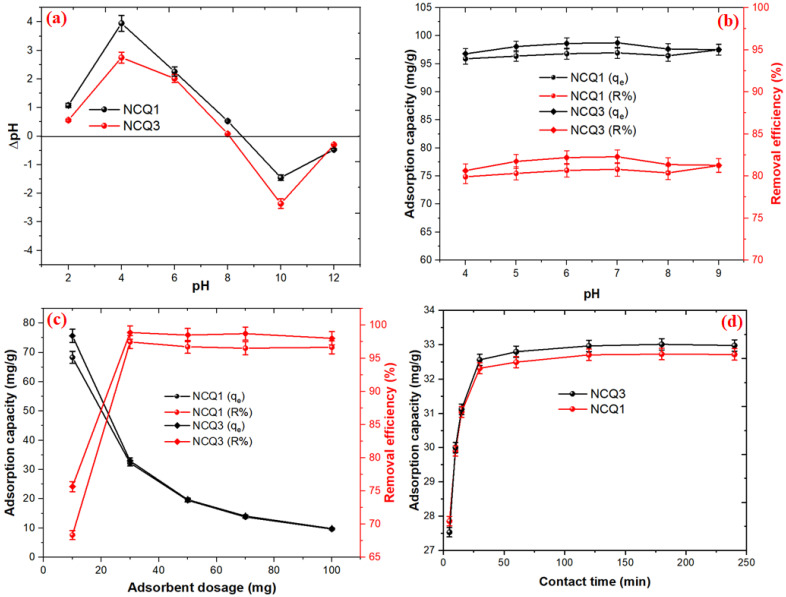
(**a**) Point of zero charges (pH_PZC_). (**b**) Effect of different parameters: pH, (**c**) adsorbent dosage, (**d**) and contact time on adsorption CV dye onto both NCQ1 and NCQ3 adsorbents.

As we know, the purple color of crystal violet molecules would fade at alkaline conditions more than pH = 9 because the positively charged CV cation is converted into a carbinol base (non-resonant) [46]. Therefore, the influence of pH on the CV adsorption onto both NCQ1 and NCQ3 adsorbents was studied at a pH range of 4–9 as shown in Figure 5a (where [CV] = 50 mg/L; dose = 30 mg; temperature = 25 °C, speed = 100 rpm, time = 24 h). It was observed that the removal efficiencies of CV adsorption using both NCQ1 and NCQ3 adsorbents were increased slightly from 95.9% to 97.0% and from 96.7% to 98.7% upon increasing pH from 4 to 7, respectively. The observed point of zero charges (pH_PZC_) for both NCQ1 and NCQ3 was 8.5 and 8.1, respectively (Figure 5b). The micro and nanoclay surfaces were positively charged at a pH less than pH_pzc_, (pH < pH_Pzc_) while at a pH higher than pH_pzc_ (pH > pH_Pzc_), both NCQ1 and NCQ3 were negatively charged. This means that ion exchange between the positive Na^+^ in the interlayer of adsorbents and cationic CV+ occurred, and the maximum adsorption capacities were 80.8 and 82.3 mg/g for both NCQ1 and NCQ3 adsorbents, respectively. In addition, below pH = 3 and above pH = 9, the dye’s disappearance may be due to the conversion of the CV cation into a carbinol base [47].

Figure 5c demonstrated that the factors that influence the amount of adsorbent in the range (10–100 mg) in the adsorption process of CV dye, when the initial CV concentration was 20.0 mg/L, were an agitation speed of 100 rpm, a contact time of 24 h, and a temperature of 25 °C. The results reveal that the removal efficiency of CV by NCQ1 and NCQ3 was rapidly increased from 68.35% to 97.46% and from 75.66% to 98.88% upon increasing the amount of the adsorbent from 10 to 30 mg, respectively. On the other hand, the adsorption capacities of NCQ1 and NCQ3 adsorbents were reduced from 68.35 to 9.66 mg/g and from 75.66 to 9.8 mg/g upon raising the amount of the adsorbent from 10 to 30 mg, respectively. Beyond 30 mg, the values of adsorption capacity were constant. The decrease in adsorption capacity may be due to two reasons. The first reason is that some of the active sites on nanoclay adsorbents were left unoccupied because the ratio between the adsorbent active site and adsorbate was very large. The second reason is increasing the amount of adsorbent led to an increase in the diffusion path length resulting from the aggregation or overlap of adsorption active sites of adsorbent, resulting in a reduction in adsorption [48,49].

Figure 5d illustrates the influence of the contact time (in the range of 5–480 min) on the equilibrium at a fixed concentration of 20 mg/L and the constant temperature of 25 °C on the adsorption capacities of the CV dye onto NCQ1 and NCQ3 micro and nanoclays. The removal efficiency was observed to be 83.6% and 82.6% within 5 min for both NCQ1 and NCQ3 adsorbents, respectively. The adsorption capacities and removal efficiency values for the CV dye were rapidly increased at the early stage of the adsorption process and nearly reached an equilibrium state after 120 min for both adsorbents. The removal efficiencies and adsorption capacities of NCQ1 and NCQ3 for the CV dye were (98.12%, 32.70 mg/g) and (98.89%, 32.96 mg/g) at 120 min, respectively. The NCQ3 exhibited slightly higher removal efficiency and adsorption capacity values than that of NCQ1, which might be due to the higher amount of alumina and silica present in the NCQ3 adsorbent compared to that in the NCQ1 adsorbent [50]. Thus, a time of 120 min was selected as the proper time for both adsorbents to ensure a complete adsorption process.

**Figure 6:**
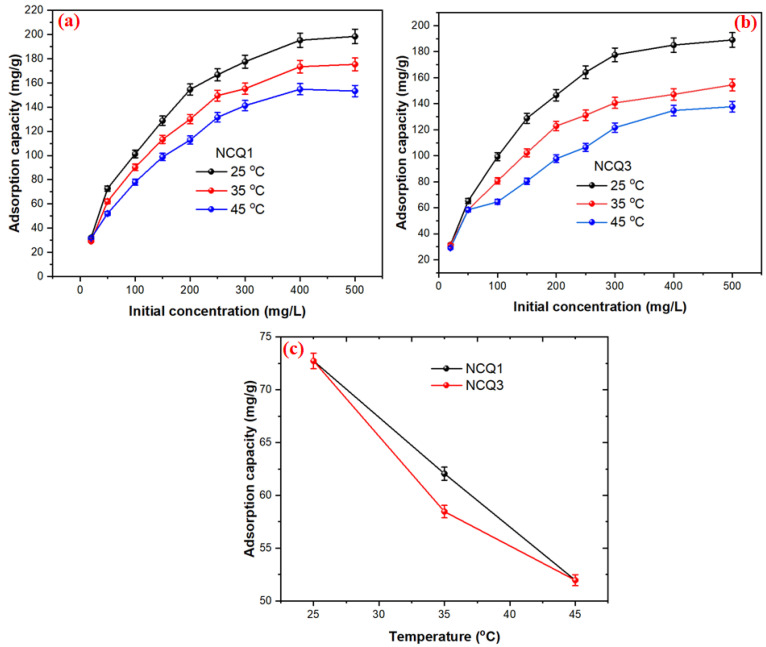
(**a**) Effect of initial CV concentration on adsorption of CV dye onto NCQ1 (**b**) and NCQ3 (**c**) Effect of temperature on adsorption of CV dye onto both NCQ1and NCQ3 adsorbents.

Figure 6a,b presents the influence of the initial CV concentrations on the removal efficiency of CV by NCQ1 and NCQ3 adsorbents at various concentrations (20 to 500 mg/L) and temperatures (25, 35 and 45 °C). The other adsorption parameters (dose = 30 mg, temperature = 25 °C, agitation speed = 100 rpm) were kept constant throughout the experiment. It is noticed that the adsorption capacities of both tested adsorbents NCQ1 and NCQ3 increased from 31.93 mg/g to 194.46 mg/g and from 31.51 mg/g to 189.06 mg/g, respectively, with the rise in the initial CV concentration from 20 to 500 mg/L at 25 °C. This increase in the adsorption capacities of NCQ1 and NCQ3 adsorbents upon increasing the initial CV concentrations is associated with the high probability of effective collisions between CV ions with the available active sites present on NCQ1 and NCQ3 adsorbents along with the increase in the driving force for mass transport [51].

The influence of various temperatures of 25, 35 and 45 °C on the CV adsorption by NCQ1 and NCQ3 adsorbents is presented in Figure 6c. The results exhibited that the removal percentage of CV ions onto NCQ1 and NCQ3 adsorbents was reduced from 72.72 mg/g to 51.96 mg/g and from 65.21 mg/g to 58.46 mg/g upon increasing the temperature from 25 to 45 °C, respectively. This indicates that the adsorption of the CV dye on both adsorbents is an exothermic process. These observations were similar to those reported by Khan et al., 2020, in which a reduction in the adsorption capacity of silico-manganese fumes of crystal violet occurred with an increase in temperature [52].

### 3.4. Adsorption Isotherm

**Figure 7:**
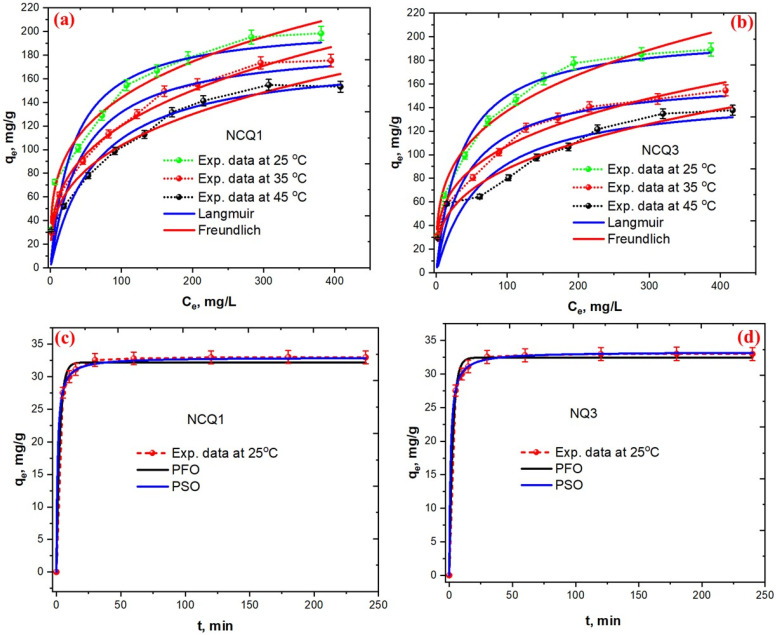
(**a**) Nonlinear isotherm adsorption models for CV dye adsorption onto NCQ1 (**b**) and NCQ3 adsorbents. (**c**) Nonlinear kinetic adsorption models for CV dye adsorption onto NCQ1 (**d**) and NCQ3 adsorbents.

To investigate the equilibrium and correlation of the residual CV dye concentration, which remains in the solution, and the adsorption capacity of both tested micro and nanoclays NCQ1 and NCQ3, data were analyzed utilizing Langmuir and Freudlich isotherm models [53]. The Langmuir isotherm model considers monolayer adsorption on the surface, which is made up of a fixed quantity of similar sites for adsorption where each site can accommodate only one CV dye molecule. Moreover, the Langmuir isotherm is utilized to decide if it is economical and effective to use the tested nanoclays for the elimination of CV dye from a water solution. The isotherm model of Freundlich states that the adsorption process happens on different surfaces. The nonlinear Langmuir and Freundlich isotherm models are mathematically stated in Equations (3) and (4) below.

(3) $$q_{e}=\frac{q_{m}K_{L}C_{e}}{1+K_{L}C_{e}}$$

(4) $$q_{e}=K_{F}\;{C_{e}}^{1/n}$$

where *q_e_* and *q_m_* are, respectively, the equilibrium and maximum adsorption capacities (mg/g); *C_e_* is the concentration of the CV dye at equilibrium (mg/L); *K_L_*, *K_F_*, and *n* refer to the Langmuir constant (L/mg), Freundlich constants, and the adsorption intensity, respectively. Equation (5) below shows the dimensionless equilibrium constant called the separation factor (*R_L_*), which is used to indicate the type of isotherm; if (0 < *R_L_* < 1) the adsorption process is favorable, while it is unfavorable if (*R_L_* < 1), irreversible if (*R_L_* = 0), and linear if (*R_L_* = 1) [52].

(5) $$R_{L}=\frac{1}{1+K_{L}C_{o}}$$

where *K_L_* is the Langmuir constant and *C_o_* is the initial CV concentration. The non-linear Langmuir and Freundlich isotherm parameters are shown in Table 4 and the data of the adsorption isotherm is shown in Figure 7a,b. The outcomes showed that the CV ion adsorption onto both NCQ1 and NCQ3 adsorbents followed the Freundlich model with a high correlation coefficient (*R*^2^), which was 0.982 and 0.973 for NCQ1 and NCQ3 adsorbents at 25 °C, respectively, suggesting that the adsorption process occurred on the surface of both tested micro and nanoclays that have different types of adsorption sites. The values of *n* were in the range of (3.602–3.059) and (3.430–3.066) for NCQ1 and NCQ3 adsorbents, respectively, indicating a favorable adsorption process. In addition, the observed high value of *K_F_* at 25 °C indicates a high adsorption amount of the CV dye onto the surface of NCQ1 and NCQ3 adsorbents. According to the range values of *R_L_* for both adsorbents, the CV dye adsorption was favorable in the tested concentration range. Based on the Langmuir model, the maximum monolayer capacities were 206.73 mg/g and 203.66 mg/g at 25 °C for the CV dye onto NCQ1 and NCQ3 adsorbents, respectively.

### 3.5. Kinetics Adsorption

To analyze the experimental data of CV adsorption and achieve the kinetic mechanism of CV dye adsorption onto NCQ1 and NCQ3 adsorbents, the nonlinear, pseudo-first-order (PFO) Equation (6) [54] and pseudo-second-order (PSO) [55] Equation (7) models were employed.

(6) $$q_{t}=q_{e}(1-e^{-k_{1}t})$$

(7) $$q_{t}=\frac{q_{e}^{2}k_{2}t}{1+q_{e}k_{2}t}$$

where *k*_1_ and *k*_2_ represent the rate constant of PFO and PSO, respectively; *q_e_* and *q_t_* represent the quantities of CV adsorbed using both selected micro and nanoclay adsorbents at equilibrium and at time *t*, respectively. The nonlinear kinetics parameters are tabulated in Table 5 and the data of the adsorption kinetics are presented in Figure 7c,d. Based on *R*^2^, the adsorption of the CV dye onto both adsorbents obey the PSO model, suggesting that the CV adsorption onto both examined micro and nanoclays are via chemical adsorption. The experimental adsorption capacities values (*q_e_*,_exp._) were observed to be close to the calculated adsorption capacities (*q_e_*,_cal._) in the case of PSO models for both adsorbents (Table 5).

### 3.6. Adsorption Thermodynamics

**Figure 8:**
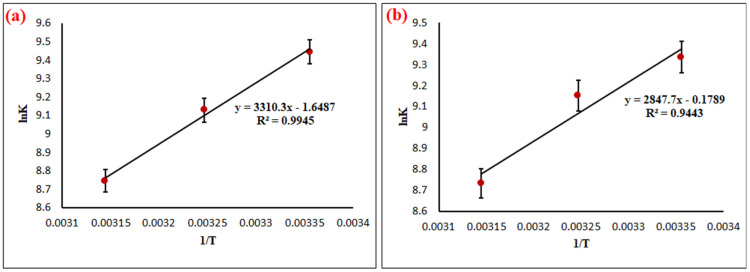
(**a**) Plot of *lnK* vs. 1/*T* for thermodynamic parameters calculation for adsorption of CV dye onto NCQ1 (**b**) and NCQ3 adsorbents.

To study the thermodynamic behavior of the CV adsorption onto both NCQ1 and NCQ3 adsorbents, the thermodynamic parameters ((Δ*G*°) Equation (8), (Δ*H*°), and (Δ*S*°) Equation (9)) were calculated using the following equations:

(8) $$\Delta G{}^{\circ}=-RT\;lnK_{e}^{o}\;$$

(9) $$lnK_{e}^{o}\;=\;-\frac{\Delta H{}^{\circ}}{RT}+\frac{\Delta S{}^{\circ}}{R}\;\left(\mathrm{Van}’t\;\mathrm{Hoff}\;\mathrm{equation}\right)\;$$

where $K_{e}^{o}\;$(L mol^−1^) represents the thermodynamic equilibrium constant that is dimensionless [56,57,58]. It is calculated by converting the units of *K_L_* (Langmuir equilibrium constant) that is given initially in L mg^−1^ into L mol^−1^ by the multiplication of the value of *K* (L mg^−1^) by 1000, subsequently making the multiplication of this result the molecular weight of the CV dye. The results of $K_{e}^{0}$ (L mol^−1^) are shown in Table 6. The Δ*H°* and Δ*S°* were calculated from the plot of *lnK_c_* versus 1/*T* as shown in Figure 8a,b. The thermodynamic parameters for CV adsorption onto both NCQ1 and NCQ3 adsorbents were summarized in Table 6. The negative Δ*G*°, Δ*H*°, and Δ*S*° values indicate the CV dye adsorption onto both NCQ1 and NCQ3 adsorbents was spontaneous, exothermic in nature, and exhibited a reduction in randomness at the solid/liquid interface during adsorption, respectively [59,60]. In addition, it was observed from Table 6 that the Δ*G°* values were reduced upon increasing the temperature from 25 °C to 45 °C, suggesting that the CV adsorption onto both adsorbents became more favorable and spontaneous at 25 °C. The Δ*H*° value for both NCQ1 and NCQ3 adsorbents was −27.52 and −23.67 kJ mol^−1^, respectively, indicating a chemical interactions mode [61].

### 3.7. Adsorption Mechanism

The mechanism of CV dye adsorption on both NCQ1 and NCQ3 adsorbents has been decided based on EDX, FTIR, and isotherm, kinetic, and thermodynamic studies. The interpretation of adsorption of CV on both NCQ1 and NCQ3 adsorbents can be mainly explained based on ion exchange. It can be observed from EDX results that K^+^ and Ca^2+^ were decreased in both NCQ1 and NCQ3 adsorbents after cationic CV adsorption and new peaks appeared for N and C, which may be a consequence of the exchange of cationic CV by K^+^ and Ca^2+^ cations. Form FTIR spectra, the peak at 3456 cm^−1^ was shifted to a lower frequency at 3452 cm^−1^ and 3340 cm^−1^ after CV adsorption on NCQ1 and NCQ3, respectively, indicating the formation of hydrogen bonds between the CV dye and the NCQ1 and NCQ3 surfaces [62,63]. The peaks in the range of 988 cm^−1^ to 524 cm^−1^ were reduced to a lower frequency (978–518 cm^−1^) after CV adsorption due to the ion exchange of the CV dye with K^+^, Na^+^, and Ca^2+^ on the surface of the nanoclay or in the interlayer [36]. The nonlinear kinetic studies showed a better fit to PSO model kinetics, indicating a chemical adsorption process. In addition, the high value of Δ*H*° for both adsorbents indicated a chemical interactions mode [58].

### 3.8. Comparison of Saudi Natural Micro and Nanoclay with Other Adsorbents

Table 7 shows the adsorption capacities of different clay adsorbents towards the CV dye. It is clear that the adsorption capacities of Saudi natural micro and nanoclays were better than other reported clay adsorbents for CV dye adsorption. Therefore, it could be concluded that the Saudi natural micro and nanoclay adsorbents have considerable potential for the elimination of the CV dye from an aqueous environment.

### 3.9. Antimicrobial Activity

From the results, it was observed that the clay samples NCQ1, NCQ3, and NCQ5 were active and showed a significant zone of inhibition. The NCQ1 sample showed better activity than the standard drug, ciplofloxacin, with zones of inhibition of 32 mm (*S. aureus*), 22 mm (*E. faecalis*), 35 mm (*K. pneumoniae*), and 35 mm (*E. coli*) whereas ciplofloxacin displayed zones of inhibition of 24 mm (*S. aureus*), 25 mm (*E. faecalis*), 14 mm (*K. pneumoniae*), and 14 mm (*E. coli*). The NCQ5 sample was found to be active against the Gram-negative bacterial strains *K. pneumoniae* (26 mm) and *E. coli* (24 mm), whereas NCQ3 showed a zone of inhibition of 10 mm against E. coil only. The literature survey reveals that clay minerals possess different biological activities, especially antimicrobial properties [66,67]. The antimicrobial properties differ with the variation in the percentage of the different elements that may be present in the clay material. It is reported that the clay with a high percentage of iron and aluminum showed better antimicrobial properties [68,69,70]. Among all the tested clay samples, the NCQ1 clay sample has the highest percentage of iron, which may be responsible for its prominent activity against most of the tested microbial strains. Sample NCQ5 had the highest percentage of aluminum and had a good percentage of iron too, which may enhance its antimicrobial activity. The results of the antimicrobial activity are shown in Table 8.

## 4. Conclusions

The present study indicates that Saudi natural clay acts as an effective adsorbent for the elimination of CV dye from aqueous solutions. Different techniques such as SEM-EDX, FTIR, XRF, XRD, and ICP-MS were applied to characterize the examined micro and nanoclay adsorbents. The results of these techniques confirmed the morphology, structure, and active sites of the micro and nanoclay adsorbents. The effects of adsorbent dosage, contact time, temperature, pH, and initial CV concentration were investigated to assess the performance of both NCQ1 and NCQ3 adsorbents for the adsorption of CV dye from aqueous solutions. The results showed that the optimized working conditions were found to be an adsorbent dose of 30 mg, a temperature of 25 °C, an equilibrium time of 120 min, and an initial CV concentration of 400 mg/L. The Freundlich isotherm model was better than the Langmuir isotherm to describe the adsorption behavior of the CV dye using both nanoclays. The adsorption of the CV dye by both adsorbents obeyed the PSO and PFO kinetic models, which indicated a chemical and physical interaction occurred between the CV dye and both nanoclay adsorbents. The maximum adsorption capacities of both NCQ1 and NCQ3 adsorbents were found to be 206.73 mg/g and 203.66 mg/g, respectively. The thermodynamic study suggested that the adsorption process is spontaneous and exothermic physisorption. Therefore, both natural micro and nanoclays are effective adsorbents for the removal of the CV cationic dye from an aqueous environment. From the antimicrobial study, it was concluded that the two clay samples with a high percentage of iron and aluminium (NCQ1 and NCQ5) showed significant activity against the tested microbial stains, hence these clays can be used in the preparation of antimicrobial films with polymeric materials.

## Figures and Tables

**Table 1:** Adsorption selectivity experiments with dye solutions.

Adsorbents	CV		MB		MG	
	Adsorption Capacity (mg/g ± SD)	Removal Efficiency (%)	Adsorption Capacity (mg/g ± SD)	Removal Efficiency (%)	Adsorption Capacity (mg/g ± SD)	Removal Efficiency (%)
NCQ1	11.92 ± 0.36	99.34	11.88 ± 0.36	99.02	11.79 ± 0.35	98.29
NCQ3	11.91 ± 0.35	99.25	11.85 ± 0.36	98.76	11.68 ± 0.35	97.34
NCQ4	11.84 ± 0.36	98.70	11.84 ± 0.36	98.68	11.61 ± 0.24	96.75
NCQ5	11.89 ± 0.35	99.15	11.96 ± 0.36	99.69	11.48 ± 0.35	95.67
NCR1	8.991 ± 0.27	74.93	11.14 ± 0.33	92.90	10.29 ± 0.31	85.79
NCY1	11.04 ± 0.33	92.04	11.61 ± 0.35	96.78	11.52 ± 0.34	96.06

**Table 2:** XRF data of chemical compositions of NCQ1.

Element	Weight, % ± SD	Element	Weight, % ± SD
SiO_2_	46.84 ± 0.50	SrO	0.0702 ± 0.0035
Al_2_O_3_	28.32 ± 0.20	MnO	0.0585 ± 0.0063
Fe_2_O_3_	13.59 ± 0.19	V_2_O_5_	0.046 ± 0.010
K_2_O	3.27 ± 0.14	ZrO_2_	0.0288 ± 0.0017
CaO	2.97 ± 0.08	BaO	0.0214 ± 0.0026
SO_3_	2.82 ± 0.09	ZnO	0.0114 ± 0.0006
MgO	1.10 ± 0.13	CuO	0.0097 ± 0.0007
TiO_2_	0.70 ± 0.03	As_2_O_3_	0.0076 ± 0.0018
Cl	0.139 ± 0.010		

**Table 3:** XRF data of chemical compositions of NCQ3.

Element	Weight, % ± SD	Element	Weight, % ± SD
SiO_2_	50.76 ± 0.50	CuO	0.101 ± 0.005
Al_2_O_3_	28.63 ± 0.20	ZnO	0.0958 ± 0.0048
Fe_2_O_3_	10.49 ± 0.17	SrO	0.0502 ± 0.0025
K_2_O	3.21 ± 0.14	V_2_O_5_	0.046 ± 0.011
MgO	2.89 ± 0.14	ZrO_2_	0.0284 ± 0.0014
CaO	2.04 ± 0.07	Cl	0.0263 ± 0.0075
TiO_2_	0.826 ± 0.041	PbO	0.0147 ± 0.0016
SO_3_	0.614 ± 0.048	BaO	0.0138 ± 0.0022
MnO	0.140 ± 0.007	NiO	0.006 ± 0.0008

**Table 4:** Nonlinear isotherms models for CV dye adsorption on both NCQ1 and NCQ3 adsorbents.

Model	CV Dye Adsorption					
	NCQ1			NCQ3		
	298 K	308 K	318 K	298 K	308 K	318 K
Langmuir						
*q_m_*, mg/g	206.73	189.71	179.48	203.66	165.60	152.54
*K_L_* (L/mg)	0.0310	0.0226	0.0154	0.0278	0.0231	0.0152
*R_L_*	0.6165	0.6883	0.7634	0.6421	0.6831	0.7664
*R* ^2^	0.8813	0.9415	0.9191	0.9445	0.9082	0.8036
Freundlich						
*K_F_*, (mg/g) (L/mg)^1/*n*^	40.093	27.358	22.997	35.813	28.183	19.622
*n*	3.6025	3.1122	3.0591	3.4303	3.3429	3.0663
*R* ^2^	0.9825	0.9822	0.9673	0.9739	0.9790	0.9586

**Table 5:** Nonlinear kinetics model for CV dye adsorption on both NCQ1 and NCQ3 adsorbents.

Adsorbent	*C_o_* (mg/L)	*q_e_*_,exp._ (mg/g ± SD)	Pseudo-First-Order			Pseudo-Second-Order		
			*q_e_*_,cal._ (mg/g ± SD)	*K*_1_ (1/min)	*R* ^2^	*q_e_*_,cal._ (mg/g ± SD)	*K*_2_ (g/mg-min)	*R* ^2^
NCQ1	20	32.71 ± 0.39	32.17 ± 0.32	0.377 ± 0.035	0.994	32.96 ± 0.08	0.033 ± 0.084	0.9997
NCQ3	20	32.97 ± 0.25	32.92 ± 0.02	0.334 ± 0.026	0.993	33.29 ± 0.08	0.029 ± 0.001	0.9997

**Table 6:** Thermodynamics factors for CV dye adsorption on both NCQ1 and NCQ3 adsorbents.

	Temperature (K)		
	298	308	318
**NCQ1**			
*K_L_* (L mol^−1^)	1.264 × 10^4^	9.220 × 10^3^	6.282 × 10^3^
∆*G*° (kJ mol^−1^)	−23.40	−23.37	−23.12
∆*H*° (kJ mol^−1^)	−27.52		
∆*S*° (J K^−1^ mol^−1^)	−13.70	-	-
*R* ^2^	0.9945	-	-
**NCQ3**			
*K_L_* (L mol^−1^)	1.134 × 10^4^	9.424 × 10^3^	6.201 × 10^3^
∆*G*° (kJ mol^−1^)	−23.13	−23.43	−23.08
∆*H*° (kJ mol^−1^)	−23.67		
∆*S*° (J K^−1^ mol^−1^)	−1.487	-	-
*R* ^2^	0.9443	-	-

**Table 7:** Comparison of the CV adsorption capacity of both NCQ1 and NCQ3 adsorbent with other clay adsorbents.

Adsorbent	Conditions	*q_m_* (mg/g)	Ref.
Tunisian Smectite Clay	*C_o_*—12.5–100 mg/L; pH—8; dose—50 g/L: time—30 min	86.54 mg/g	[2]
Moroccan pyrophyllite	*C_o_*—5–20 mg/L; pH—6.5; *T*—293 K; dose—1g/L: time—20 min	12.5 mg/g	[6]
Kaolin	*C_o_*—10–100 mg/L; pH—7; *T*—295 K; dose—1 g/L: time—30 min	47.27	[20]
Surfactant modified bentonite clay	*C_o_*—400 μmol/L; *T*—303K; pH—9; dose—0.1 g/L: time—240 min	365.11 μmol/g	[63]
Halloysite	*C_o_*—20–400 mg/L; pH—7; *T*—298K; dose—1 g/L: time—240 min	194.5	[64]
KG-g-PMETAC/MMT	*C_o_*—100 mg/L; pH—7; *T*—298 K; dose—50 mg; time—5 h	137.77	[65]
NCQ1 and NCQ3	*C_o_*—20–500 mg/L; pH—7; *T*—298 K; dose—30 mg: time—120 min	206.73 and 203.66	This study

**Table 8:** Antimicrobial activity of different clay samples against Gram-positive and Gram-negative bacterial strains.

Samples	Zone of Inhibition (Mean in mm ± SD)			
	Gram-Positive Bacterial Strains		Gram-Negative Bacterial Strains	
	*S. aureus*	*E. faecalis*	*K. pneumoniae*	*E. coli*
NCQ1	32 ± 1.24 ***	22 ± 1.12 **	35 ± 1.34 ***	35 ± 1.32 ***
NCQ3	0	0	0	10 ± 0.46 *
NCQ4	0	0	0	0
NCQ5	10 ± 0.36 *	10 ± 0.36 *	26 ± 1.10 **	24 ± 1.08 **
NCR1	0	0	0	0
NCY1	0	0	0	0
NC	0	0	0	0
P.C	22 ± 1.02 **	25 ± 1.04 **	14 ± 0.54 *	14 ± 0.56 *

Data were analyzed by one way ANOVA followed by Dunnett’s ‘*t*-test (*n* = 3).* *p* < 0.05, ** *p* < 0.01 and *** *p* < 0.001 significantly different from negative control; NC: Negative control (polyvinyl alcohol); P.C: Positive control (ciprofloxacin).

## Data Availability

The data presented in this study are available on request from the corresponding author.
